# Elevated expression of HDAC6 in clinical peritoneal dialysis patients and its pathogenic role on peritoneal angiogenesis

**DOI:** 10.1080/0886022X.2020.1811119

**Published:** 2020-08-30

**Authors:** Yingfeng Shi, Jun Ni, Min Tao, Xiaoyan Ma, Yi Wang, Xiujuan Zang, Yan Hu, Andong Qiu, Shougang Zhuang, Na Liu

**Affiliations:** a Department of Nephrology, Shanghai East Hospital, Tongji University School of Medicine, Shanghai, China; b Department of Immunology and Microbiology, Shanghai Institute of Immunology, Shanghai Jiao Tong University School of Medicine, Shanghai, China; c Department of Nephrology, Baoshan Branch of Shanghai First People’s Hospital, Shanghai, China; d Department of Nephrology, Shanghai Songjiang District Central Hospital, Shanghai, China; e School of Life Science and Technology, Advanced Institute of Translational Medicine, Tongji University, Shanghai, China; f Department of Medicine, Rhode Island Hospital and Alpert Medical School, Brown University, Providence, RI, USA

**Keywords:** Histone deacetylase 6, peritoneal fibrosis, peritoneal dialysis, angiogenesis, vascular endothelial growth factor

## Abstract

Peritoneal dialysis (PD) is an important renal replacement therapy for end-stage renal disease (ESRD) patients. However, its complications, such as peritoneal fibrosis (PF) and angiogenesis can cause ultrafiltration failure and PD termination. Histone deacetylase 6 (HDAC6) has been demonstrated to be involved in PF. However, its underlying role in peritoneal angiogenesis is still unknown and clinical value needs to be explored. In this study, we analyzed the expression of HDAC6 in the peritoneum from patients with non-PD and PD-related peritonitis and dialysis effluent from stable PD patients. Our study revealed that HDAC6 expressed highly in the peritoneum with peritonitis and co-stained with α-smooth muscle actin (α-SMA), a biomarker of the myofibroblast. And the level of HDAC6 in the dialysate increased with time and positively correlated with transforming growth factor-β1 (TGF-β1), interleukin-6 (IL-6) and vascular endothelial growth factor (VEGF), and negatively with cancer antigen 125 (CA125). *In vitro*, blockading HDAC6 with a selective inhibitor tubastatin A (TA) or silencing HDAC6 with a small interfering RNA (siRNA) prominently decreased IL-6-stimulated VEGF expression in cultured human peritoneal mesothelial cells (HPMCs), and inhibited proliferation and vasoformation of human umbilical vein endothelial cells (HUVECs). TA or HDAC6 siRNA also suppressed the expression of Wnt1, β-catenin, and the phosphorylation of STAT3 in IL-6-treated HPMCs. In summary, HDAC6 inhibition protects against PD-induced angiogenesis through suppression of IL-6/STAT3 and Wnt1/β-catenin signaling pathway, subsequently reducing the VEGF production and angiogenesis. It could become a new therapeutic target or forecast biomarker for PF, inflammation, and angiogenesis in the future.

## Introduction

Peritoneal dialysis (PD) is an effective strategy of renal replacement therapy for patients with end-stage renal disease (ESRD), characterized by multiple advantages including protection of residual renal function, hemodynamic stability, home care [1,2]. However, sustained exposure to non-biocompatible peritoneal dialysis fluid (PDF) and PD patients’ specific microenvironment, such as peritoneal inflammation, fibrosis, and angiogenesis can destroy normal peritoneal structure and function, leading PD termination [3,4]. Therefore, the development of biomarkers on early diagnosis and treatment on the aforementioned pathological changes are urgent problems that are ultimately beneficial to patients with long-term PD.

Peritoneal angiogenesis is one of the common peritoneal structural changes for PD patients and also the principal mechanism for ultrafiltration failure (UFF) [5]. The expanded vascular network increases effective surface area exchange and decreases the glucose-driven osmotic pressure and ultrafiltration. It results from increased production of vascular endothelial growth factor (VEGF) and other cytokines that stimulate the formation of new capillaries and vasculopathy [5]. Impaired peritoneal mesothelial cells (PMCs) are the main source of VEGF secretion due to the continuous stimulation by non-biocompatible PDF [5–7]. And VEGF production is mainly regulated by IL-17 [8] and IL-6 dependent signaling pathways [9,10] or Wnt/β-catenin signaling pathway [11]. Several types of research also report the cooperation between vascularization and peritoneal fibrosis (PF) [12,13]. Moreover, clinical data from our previous researches and others have shown that VEGF was highly expressed in human dialysate effluent and significantly correlated with the number of vessels in UFF patients [14–16].

Besides VEGF, other cytokines and growth factors have also been detected and proved to associate with peritoneal injury, including fibrogenesis (PAI-1, CTGF, TGF-β, procollagen peptides), inflammation (IL-6, TNF-α), tissue remodeling (CCL18, HA, MMP-2) and mesothelial cell mass represented by CA125 [17]. Among them, IL-6 is the most important and original proinflammatory factor during the peritoneal inflammatory response, and as a medium linking inflammation and angiogenesis [18]. Furthermore, IL-6 has the ability to promote the epithelial-to-mesenchymal transition (EMT) process and production of proangiogenic factors in cultured human peritoneal mesothelial cells (HPMCs) [19,20]. CA125, a high molecular weight (220 kDa) glycoprotein, is mainly produced by PMCs [21]. Based on this, CA125 in peritoneal effluent can be regarded as a marker for the mesothelial cell mass and reflects the condition of the peritoneal membrane in stable PD patients [21–23]. So, expression levels of these biomolecules can be used as predicted biomarkers to estimate the progression of peritoneal injury for long-term PD patients to a certain extent. Recently, it has been demonstrated that epigenetic enzyme can regulate the progression of PF by modifying a series of signaling pathway proteins and transcription factors [14,24,25].

Histone acetylation and deacetylation are the predominant form in epigenetic fields and are modified by histone acetylase and deacetylase [26–28]. Histone deacetylase 6 (HDAC6) belongs to the class II HDAC family and participates in multiple physiological and pathological processes. Numerous studies have proved the pathogenic role of HDAC6 involved in various fibrotic diseases, such as renal fibrosis [29], pulmonary fibrosis [30], and hepatic fibrosis [31]. Inhibition of HDAC6 effectively attenuates tissue fibrogenesis and improves tumor deterioration by regulating cell cycle, proliferation, and migration [32]. Our previous publication is the first to find that HDAC6 also contributes to PF in a 4.25% high glucose PDF-induced mouse model through two major mechanisms, EMT of PMCs and inflammation [33]. However, the underlying regulatory mechanism of HDAC6 in VEGF production and peritoneal angiogenesis is still unknown, and its clinical value needs to be further explored. In this current study, we detected the expression level of HDAC6 in both peritoneum and dialysis effluent from clinical PD patients and verified whether HDAC6 regulates the VEGF production from HPMCs and has an impact on vasoformation of endothelial cells.

## Materials and methods

### Antibodies and reagents

Tubastatin A was purchased from Selleckchem (Houston, TX). Antibodies to HDAC6 (#7612), STAT3 (#9139), Phospho-STAT3 (#9138) and α-Tubulin (#3873) were purchased from Cell Signaling Technology (Danvers, MA). Antibody to Collagen I (A2) (sc-28654) and GAPDH (sc-32233) were purchased from Santa Cruz Biotechnology (San Diego, CA). Antibody to Wnt1 (#35866) was purchased from Rockland (Plymouth, MA). Antibody to β-catenin (610154) was purchased from BD Transduction Laboratories (Franklin Lake, NJ). HDAC6, TGF-β1, IL-6, VEGF, CA125 enzyme-linked immunosorbent assay (ELISA) kits, and IL-6 protein were purchased from R&D Systems (Minneapolis, MN). HDAC6 siRNA was purchased from GenePharma (Shanghai, China). Lipofectamine 2000 was purchased from Invitrogen (Grand Island, NY). Matrigel was purchased from Corning (Tewksbury, MA). Antibody to α-SMA (A2547) and all other chemicals were obtained from Sigma-Aldrich (St. Louis, MO).

### Clinical sample collection and ethics statement

To determine HDAC6 expression in the peritoneum samples from patients with non-PD and PD-related peritonitis, we collected peritoneal tissue during operations to catheterization initiation (*n* = 6) and refractory peritonitis-induced catheter migration (*n* = 6) at Shanghai East Hospital affiliated with Tongji University and completed co-immunofluorescence staining of HDAC6 and α-SMA. We also collected the PD effluents in diverse PD time, respectively, time ≤ 1 year (*n* = 24), 1 < time ≤ 3 years (*n* = 15) and time > 3 years (*n* = 32) at three PD centers, Shanghai East Hospital, Shanghai Baoshan Branch of First People’s Hospital and Shanghai Songjiang District Central Hospital from July 2017 to December 2019. The inclusion criterion is the continuous ambulatory peritoneal dialysis (CAPD) patients who conduct regular peritoneal equilibration test (PET) using PD solution (Lactate-G1.5% or 2.5%), without peritonitis, severe heart failure, unstable angina, active liver disease, trauma and operation within the past month. Unpolluted dialysis effluents are collected during PET and the supernatant is detected by immunoblotting assay and ELISA kits after high-speed centrifugation. This study was approved by the Medical Ethics Committee of Shanghai East Hospital, Shanghai Baoshan Branch of First People’s Hospital, and Shanghai Songjiang District Central Hospital and was conducted in accordance with the Declaration of Helsinki. Written informed consent was obtained from each patient. And we have obtained the registration number from the Chinese Clinical Trial Register (ChiCTR): ChiCTR1800014672.

### Mesothelial cell culture

Primary HPMCs (American Type Culture Collection, ATCC; Rockville, MD) were cultured in Dulbecco’s modified Eagle’s medium (DMEM) with F12 containing 10% fetal bovine serum (FBS), 1% penicillin and streptomycin in an atmosphere of 5% CO_2_ and 95% air at 37 °C. We passed the primary cells for three generations, obtained a stable phenotype, and then started the formal experiments. To determine the effect of HDAC6 inhibition on VEGF production induced by IL-6, HPMCs were starved for 24 h with 0.5% FBS in DMEM/F12 and then exposed to IL-6 (100 ng/ml) for 48 h in the presence or absence of different doses of TA (5, 10, and 20 µM) before cell harvesting. All of the *in vitro* experiments were repeated at least three times.

### Endothelial cell culture and tube formation assay

Human umbilical vein endothelial cell (HUVEC) line was obtained from ATCC (Rockville, MD). For the tube formation assay, Matrigel was poured onto a 24-well plate and solidified at 37 °C for 30 min. Endothelial cells were seeded onto the Matrigel and cultured in high glucose medium with or without 10% (vol/vol) pre-collected cell culture media from HPMCs treated as described above. After 12 h, capillary networks of tubes formed by HUVEC were photographed and quantitatively analyzed for total segment length by ImageJ software. All of the *in vitro* experiments were repeated at least three times.

### ELISA analysis

ELISA detection of HDAC6, TGF-β1, IL-6, VEGF, CA125 protein in human PD effluents, and VEGF in cell culture media of HPMCs were performed following the manufacturer’s instructions.

### Immunoblot analysis

Immunoblot analysis was conducted as described previously [34]. The densitometry analysis of immunoblot results was conducted using ImageJ software.

### Immunofluorescence staining

Immunofluorescence staining was carried out according to the procedure described in our previous study [35]. FFPE sections (3 μm) were rehydrated and incubated with primary antibodies against α-SMA (1:100) or HDAC6 (1:200) and then Texas Red- or FITC-labeled secondary antibodies (Invitrogen).

### siRNA transfection

The small interfering (si) RNA oligonucleotides targeted especially for HDAC6 was used to downregulate HDAC6 level in cultured HPMCs. HPMCs were seeded to 30–40% confluence in antibiotic-free medium and grown for 24 h, and then were transfected with HDAC6 siRNA (50 nmol) with lipofectamine 2000 according to the manufacturer’s instructions. In parallel, scrambled siRNA (50 nmol) was used as a control for off-target changes in HPMCs. After transfection for 24 h, cells were treated with IL-6 (100 ng/ml) for an additional 48 h before being harvested for further experiments.

### Statistical analysis

All the experiments were conducted at least three times. Data depicted in graphs represented the mean ± SEM for each group. In the case of nonparametric data distribution medians with interquartile range (IQR) were presented. The univariate analysis of variance (ANOVA) was used to measure the data among the groups or a Kruskal–Wallis test in case of nonparametric data distribution. Multiple means were compared using Turkey’s test. The differences between the two groups were determined by Student’s *t*-test. A statistically significant difference between mean values was marked in each graph. As for data with non-normal distribution, Spearman’s rank tests were utilized to assess the correlation between two variables. *p* < 0.05 was considered significant. The statistical analyses were conducted by using IBM SPSS Statistics 20.0 (Version X; IBM, Armonk, NY).

## Results

### HDAC6 is highly expressed in peritoneum and dialysis effluent from PD patients

**Figure 1.**
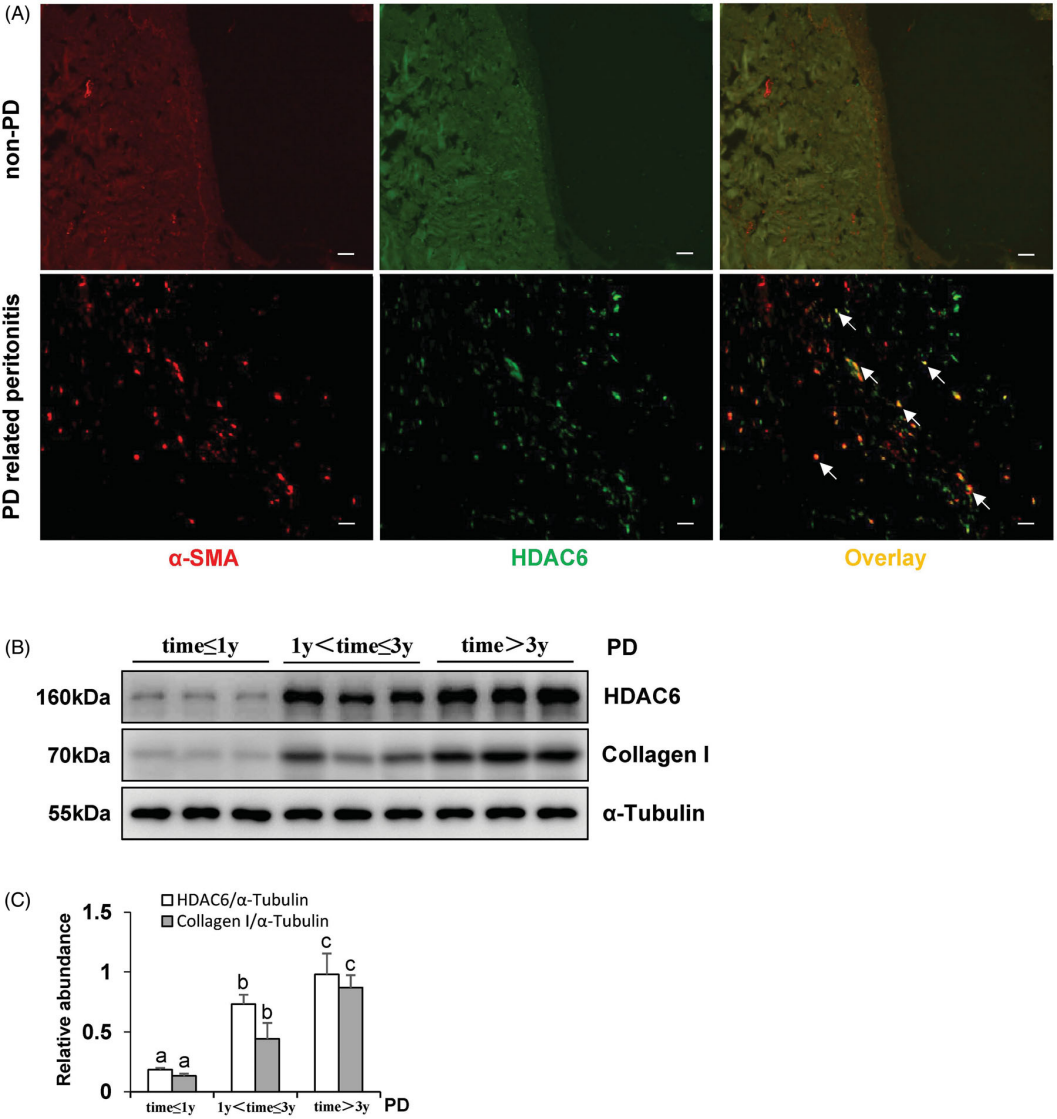
HDAC6 is highly expressed in peritoneum and dialysis effluent from PD patients. (A) Immunofluorescence photomicrographs illustrate co-staining of α-SMA and HDAC6 in the peritoneum from patients with non-PD and PD-related peritonitis. And HDAC6 was co-expressed with α-SMA-positive cells (white arrows). (B) Human PD effluents were subjected to immunoblot analysis with antibodies against HDAC6, Collagen I and α-Tubulin. (C) Expression levels of HDAC6 and Collagen I were quantified by densitometry and normalized with α-Tubulin. Data are represented as the mean ± SEM. Means with different superscript letters are significantly different from one another (*p* < 0.05). All scale bars = 20 μm.

In order to detect the expression levels of HDAC6 in the peritoneum with clinical PD patients, we collected peritoneum samples from patients who were under the surgery operations in catheterization initiation and peritonitis-induced catheter migration at Shanghai East Hospital affiliated with Tongji University. As shown in Figure 1(A), there was no thickened peritoneum, and immunofluorescence of HDAC6 or α-SMA in the tissue from non-PD patients started with catheterization. While the peritoneum from PD-related peritonitis patients showed a high expression of HDAC6 which were co-expressed with α-SMA-positive cells. Then, we collected human dialysis effluent samples of stable PD patients in diverse dialysis duration and determined the expression levels of HDAC6 and Collagen I. Clinical characteristics of these PD patients were shown in Table 1, and there was no significant statistical difference in dialysis duration comparison except for creatinine. Immunoblotting identification of dialysis effluent from different PD duration showed the elevated expression levels of HDAC6 and Collagen I in long-term PD patients, while the very low levels in dialysis effluent from new PD patients (Figure 1(B,C)). Taken together, these results indicate that HDAC6 represents abnormal high expression which is associated with peritonitis and long-term exposure injury by traditional high glucose peritoneal dialysate.

**Table 1.** Clinical characteristic of the PD patients.

Variables	Time ≤ 1 year	1 < Time ≤ 3 years	Time > 3 years	*p*-value
Number	24	15	32	
PD time (months)	1 (1–7)	24 (22–29)	48 (43–66)	
Age (years)	65.4 ± 10.5	66.3 ± 11.9	61.9 ± 13.1	0.404
Male (%)	16 (66.7%)	9 (60.0%)	16 (50.0%)	0.449
BMI (kg/m^2^)	22.9 ± 2.8	23.4 ± 3.6	25.6 ± 3.2	0.033
Drink (%)	1 (4.2%)	1 (6.7%)	2 (6.3%)	0.928
Smoke (%)	2 (8.3%)	2 (13.3%)	2 (6.3%)	0.718
Peritonitis (%)	1 (4.2%)	0 (0.0%)	5 (15.6%)	0.130
Serum albumin (%)	30.1 ± 4.9	31.1 ± 4.1	30.9 ± 6.1	0.791
TC (mmol/L)	4.1 (3.6–5.0)	3.5 (3.0–4.6)	4.0 (3.1–4.4)	0.261
TG (mmol/L)	1.7 (1.0–2.3)	1.3 (0.8–2.0)	1.9 (1.0–3.0)	0.297
HDL-C (mmol/L)	0.9 (0.8–1.0)	0.9 (0.7–1.2)	0.9 (0.6–1.0)	0.622
LDL-C (mmol/L)	2.4 (1.6–3.1)	2.0 (1.7–2.8)	2.0 (1.7–2.5)	0.688
Cr (μmol/L)	572.9 (448.1–765.7)	809.0 (684.8–921.0)	973.9 (721.2–1117.0)	<0.001
BUN (mmol/L)	18.4 (13.0–22.7)	15.2 (11.8–20.0)	18.5 (15.7–24.4)	0.265
Sodium (mmol/L)	140.3 ± 2.4	139.4 ± 4.5	139.4 ± 2.7	0.520
Potassium (mmol/L)	3.9 ± 0.8	4.1 ± 0.8	4.0 ± 0.8	0.663
Calcium (mmol/L)	2.1 ± 0.3	2.3 ± 0.2	2.3 ± 0.2	0.021
Phosphorus (mmol/L)	1.5 ± 0.3	1.5 ± 0.5	1.8 ± 0.6	0.061
Hypertension (%)	24 (100.0%)	15 (100.0%)	29 (90.6%)	0.148
Dyslipidemia (%)	6 (25.0%)	5 (33.3%)	17 (53.1%)	0.089
Diabetes mellitus (%)	10 (41.7%)	9 (60.0%)	11 (34.4%)	0.252
ACEI/ARB (%)	15 (62.5%)	12 (80.0%)	20 (62.5%)	0.445
CCB (%)	22 (91.7%)	14 (93.3%)	24 (75.0%)	0.133
Lipid-lowering drugs (%)	2 (8.3%)	5 (33.3%)	12 (37.5%)	0.041
Insulin (%)	8 (33.3%)	7 (46.7%)	9 (28.1%)	0.456

The continuous variables are reported as means ± SD and categorical variables are presented as percentages. In case of nonparametric data distribution medians with inter quartile range (IQR) are presented. Abbreviations. BMI: body mass index; TC: total cholesterol; TG: triglyceride; HDL-C: high density lipoprotein cholesterol; LDL-C: low density lipoprotein cholesterol; Cr: creatinine; BUN: blood urea nitrogen; ACEI/ARB: angiotensin converting enzyme inhibitor/ angiotensin receptor blocker; CCB: calcium channel blocker.

### HDAC6 positively correlates with enhanced expression of TGF-β1, IL-6, VEGF and negatively with CA125 in dialysis effluent of PD patients

**Figure 2.**
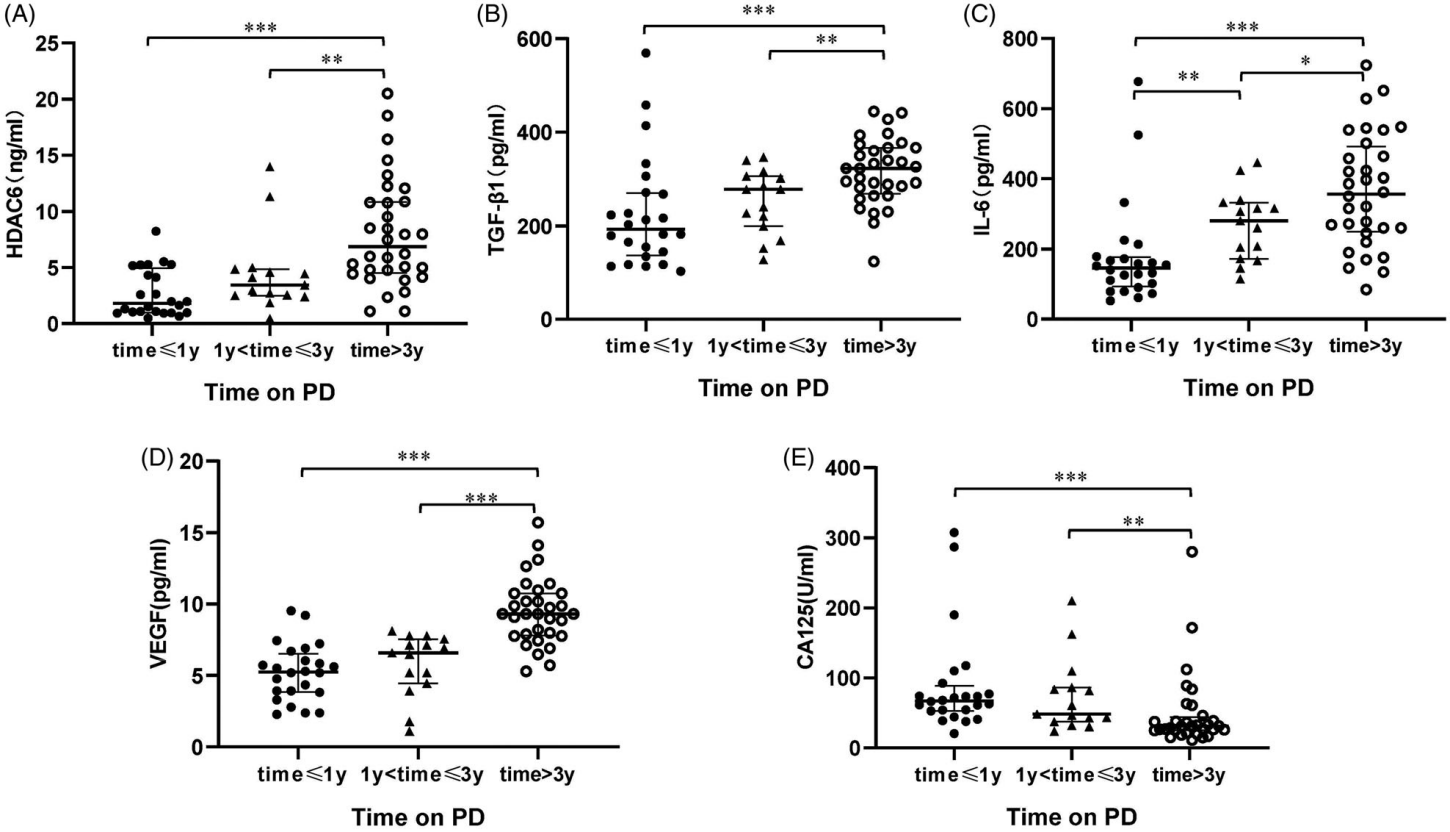
Levels of HDAC6 and cytokines in dialysis effluent according to ELISA kits. The dialysis effluents from 71 PD patients were subjected to the ELISA, as described under Materials and Methods, and divided into three PD time points, time ≤ 1 year (*n* = 24), 1 < time ≤ 3 years (*n* = 15) and time > 3 years (*n* = 32). The expression levels of HDAC6 (A), TGF-β1 (B), IL-6 (C), VEGF (D), and CA125 (E) were indicated in each group. Data are represented as the medians with inter quartile range (IQR). *0.01 ≤ *p* < 0.05; **0.001 ≤ *p* < 0.01; ***p < 0.001.

**Figure 3.**
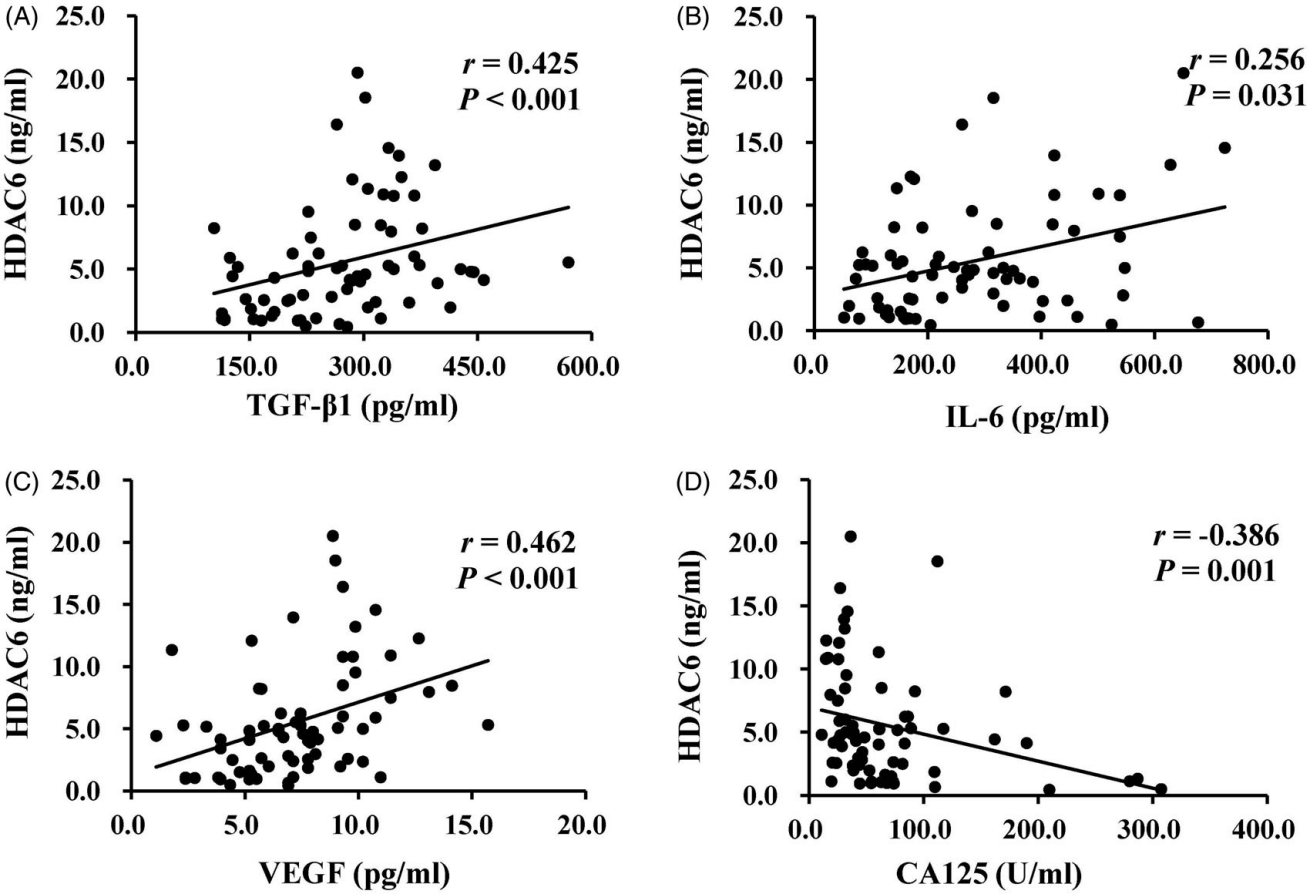
HDAC6 positively correlates with enhanced expression of TGF-β1, IL-6, VEGF, and negatively with CA125 in dialysis effluent of PD patients. Correlation analyses were conducted between HDAC6 and TGF-β1 (A), HDAC6 and IL-6 (B), HDAC6 and VEGF (C), as well as HDAC6 and CA125 (D).

Several reports have indicated that dialysate TGF-β1, IL-6, VEGF can be used to predict peritoneal membrane damage under the status of fibrosis, inflammation, and angiogenesis, respectively [17,21,36]. The expression of CA125 indicates the mesothelial cell mass lining the peritoneal membrane [21,36], as a protective indicator for PD patients. Thus, we used ELISA kits to detect the levels of HDAC6 and multiple growth factors and cytokines, such as TGF-β1, IL-6, VEGF, and CA125, in dialysis effluent from different PD duration. Results from Figure 2 showed a significantly elevated expression of HDAC6, TGF-β1, IL-6, VEGF, and a decrease of CA125 level in PD patients’ dialysis effluent with longer dialysis duration. Further correlation analyses of the above molecules in Figure 3 indicated that HDAC6 was positively correlated with enhanced expression of TGF-β1 (*r* = 0.425, *p* < 0.001), IL-6 (*r* = 0.256, *p* = 0.031), VEGF (*r* = 0.462, *p* < 0.001) and negatively with CA125 (*r* = −0.386, *p* = 0.001). These data suggest that HDAC6 may be a clinical noninvasive biomarker in dialysis effluent to forecast peritoneal membrane status and prognosis of long-term PD patients.

### Blockade of HDAC6 with TA or siRNA abrogates endothelial cell angiogenesis by inhibition of VEGF production in human umbilical vein endothelial cell

Peritoneal angiogenesis is an important pathological process for CAPD patients and is usually associated with UFF [4,5]. VEGF is a well-known angiogenetic growth factor and is mainly produced by impaired PMCs [5–7]. Here we evaluated the potential role of HDAC6 in mediating the formation of angiogenesis by examining the effect of HDAC6 inhibition on the secretion of VEGF in HPMCs, and vasoformation in human umbilical vein endothelial cell (HUVEC).

**Figure 4.**
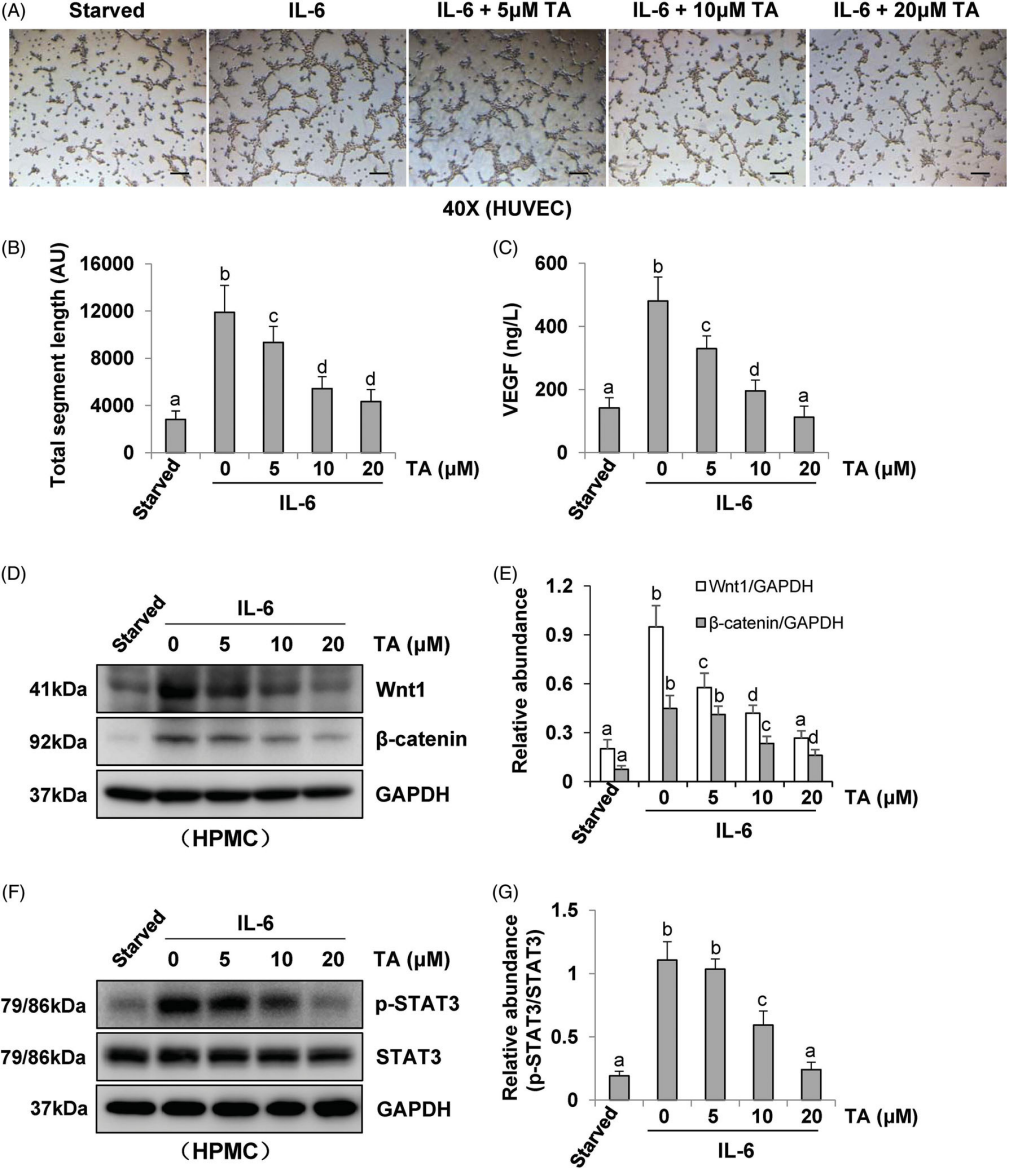
Blockade of HDAC6 with TA treatment prevents vasoformation in human umbilical vein endothelial cell. (A) Photomicrographs show capillary networks of tubes formed by HUVEC. (B) Quantitative analysis of total segment length. (C) VEGF ELISA kit was used to detect the levels of VEGF in cell culture media of HPMCs with different concentrations of TA. (D, F) Serum-starved HPMCs were pretreated with various concentrations of TA (51,020 μM) and then exposed to IL-6 (100 ng/ml) for 48 h. Cell lysates were subjected to immunoblot analysis with specific antibodies against Wnt1, β-catenin, p-STAT3, STAT3, and GAPDH. (E) Expression levels of Wnt1 and β-catenin were quantified by densitometry and normalized with GAPDH. (G) Expression level of p-STAT3 was quantified by densitometry and normalized with STAT3. Data are represented as the mean ± SEM. Means with different superscript letters are significantly different from one another (*p* < 0.05). All scale bars = 200 μm.

**Figure 5.**
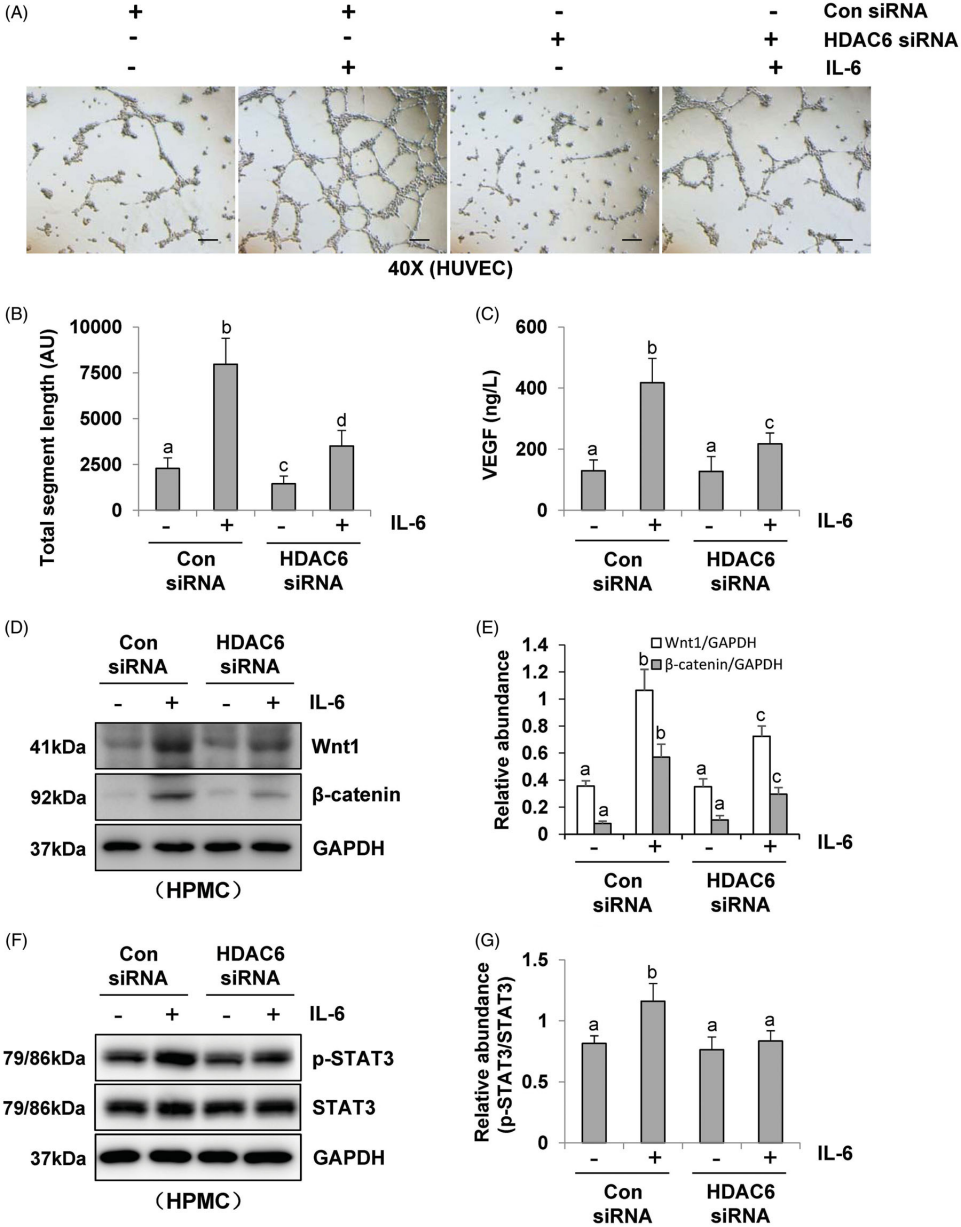
Blockade of HDAC6 with siRNA transfection prevents vasoformation in human umbilical vein endothelial cell. (A) Photomicrographs show capillary networks of tubes formed by HUVEC. (B) Quantitative analysis of total segment length. (C) VEGF ELISA kit was used to detect the levels of VEGF in cell culture media of HPMCs with HDAC6 siRNA transfection. (D, F) Serum-starved HPMCs were pretreated with HDAC6 siRNA and then exposed to IL-6 (100 ng/ml) for 48 h. Cell lysates were subjected to immunoblot analysis with specific antibodies against Wnt1, β-catenin, p-STAT3, STAT3 and GAPDH. (E) Expression levels of Wnt1 and β-catenin were quantified by densitometry and normalized with GAPDH. (G) Expression level of p-STAT3 was quantified by densitometry and normalized with STAT3. Data are represented as the mean ± SEM. Means with different superscript letters are significantly different from one another (*p* < 0.05). All scale bars = 200 μm.

Incubation of HUVEC in the presence of pre-collected cell culture media from IL-6‐stimulated HPMCs dramatically increased endothelial cell tube formation, which was inhibited by the conditioned medium from HPMCs treated with TA (Figure 4(A)). Quantitative data of endothelial cell tube formation were presented in Figure 4(B) that conditioned medium of TA treatment decreased the total segment length in HUVEC tube formation. Moreover, HPMCs medium associated with HDAC6 siRNA also inhibited endothelial cell tube formation and decreased the total segment length (Figure 5(A,B)). Given that VEGF is a potent angiogenic factor in peritoneal angiogenesis, we further examined the effect of HDAC6 blockade on the production of VEGF in HPMCs. As shown in Figure 4(C), IL-6 stimulation increased the level of VEGF in cultured HPMCs, while TA treatment dose-dependently inhibited this response. Similarly, HDAC6 siRNA suppressed IL-6 stimulated VEGF production (Figure 5(C)). In contrast, the medium from starved HPMCs in the absence of IL-6 stimulation only contained a minimum amount of VEGF. In conclusion, these results demonstrate that HDAC6 activation is required for VEGF production and endothelial cell tube formation, and suggest that HDAC6 is involved in the process of peritoneal angiogenesis during PD.

**Figure 6.**
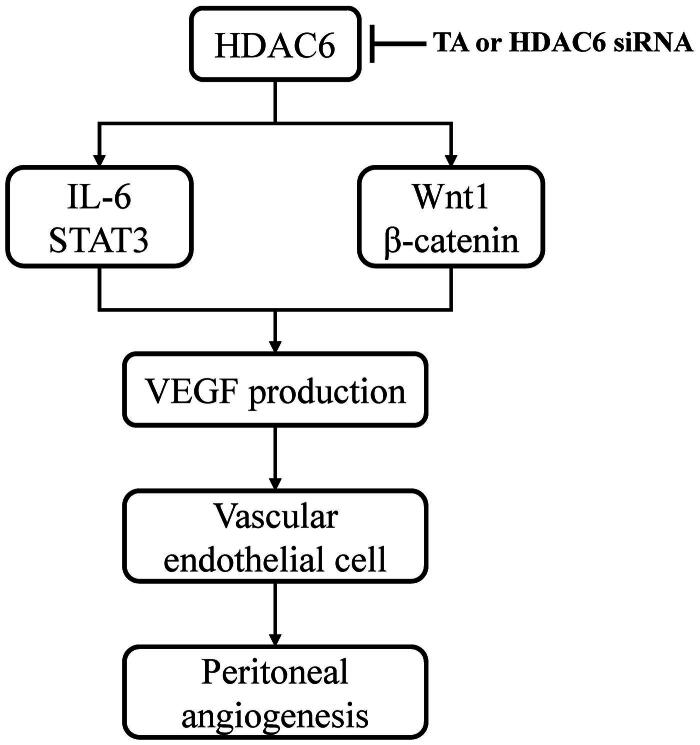
Mechanisms of HDAC6 inhibition-elicited attenuation of PD-induced angiogenesis. HDAC6 inhibition protects against PD-induced angiogenesis through at least two mechanisms: inhibition of IL-6/STAT3 signaling pathway and suppression of Wnt1/β-catenin signaling pathway, subsequently reducing the production of VEGF and angiogenesis of vascular endothelial cell.

It was reported that IL-6/STAT3 and Wnt/β-catenin signaling pathways were important angiogenic pathways involved in peritoneal angiogenesis [11,18]. To further understand the mechanism of HDAC6 in peritoneal angiogenesis, we examined the effect of TA or siRNA specifically targeting HDAC6 on IL-6-treated HPMCs. As shown in Figure 4(D–G), IL-6 significantly upregulated the expression of Wnt1 and β-catenin and increased the phosphorylation of STAT3. Administration of TA inhibited the upregulation of Wnt1, β-catenin, and p-STAT3 in a dose-dependent manner. HDAC6 siRNA also had similar inhibitory effects (Figure 5(D–G)). Collectively, these data suggest that HDAC6 inhibition protects against PD-induced angiogenesis through at least two mechanisms: inhibition of IL-6/STAT3 signaling pathway and suppression of Wnt1/β-catenin signaling pathway, subsequently reducing the production of VEGF and angiogenesis of vascular endothelial cell (Figure 6).

## Discussion

Considering the significant pathological role of HDAC6 in peritoneal injury, we further detected its expression level in clinical PD patients and explored specific regulation mechanisms on peritoneal angiogenesis in this study. We found that HDAC6 was highly expressed in the peritoneum from patients with PD-related peritonitis and the dialysis effluent of PD patients. It was upregulated in a time-dependent manner and positively correlated with the levels of TGF-β1, IL-6, and VEGF, and negatively with CA125. Moreover, *in vitro* data indicated that HDAC6 increased VEGF production and endothelial angiogenesis by regulating IL-6/STAT3 and Wnt1/β-catenin signaling pathways. On this basis, we speculated that HDAC6 might be a new therapeutic target or forecast biomarker for PF, inflammation, and angiogenesis in the future.

The current study of dialysate biomarkers in PD has been based on pathological mechanisms related to the disease course of PD, such as peritoneal membrane remodeling, inflammation, and angiogenesis. CA125, a previously well-discussed candidate biomarker, is rapidly applied to estimate mesothelial cell mass [21,37]. Effluent IL-6 is an independent predictor for poor outcome in patients starting dialysis treatment [21,38]. VEGF can be produced locally and related to the peritoneal transport of low-molecular-weight solutes [16]. Although the TGF-β1-VEGF-A pathway has been shown to play a vital role in neoangiogenesis with PF in animal models [15], evidence about the fibrosis predictor of TGF-β1 in the dialysate of human PD is still lacking. Our study showed that the expression level of TGF-β1 in the effluent of PD patients was upregulated in a time-dependent manner. And the expression level of HDAC6 was positive with TGF-β, IL-6, and VEGF, and negative with CA125. This suggests that dialysate HDAC6 could become an epigenetic biomarker for predicting the degree of multiple peritoneal injuries. However, the source of HDAC6 in the dialysis effluent remains unclear. We speculated that it may be secreted from impaired peritoneal mesothelial which is under the mesothelial-mesenchymal transition and expresses a myofibroblast phenotype. Immunofluorescence results from our present and previous study supported this assumption that HDAC6 was mainly expressed in the mesothelial layer of the injured peritoneum and co-expressed with α-SMA, a myofibroblast marker [33].

It has been reported that peritoneum under inflammatory status can induce angiogenesis [18]. A previous study showed that exposure of HPMCs to dialysate effluent containing increased levels of TGF-β and interleukins obtained during peritonitis led to a dose-dependent VEGF induction through activating VEGF promoter region with high-affinity binding sites for transcription factor c-Fos [39]. Another study from 104 samples of PD effluent collected during acute peritonitis verified correspondence between neutrophil counts and a broad range of solute parameters such as interleukins. Interleukins may be a major inducer for angiogenesis [40]. Of them, IL-6 is a cytokine, which involves in peritoneal acute-phase inflammation reaction and plays a vital role in the permeability of endothelium [18,41]. Because transducer and activator of transcription 3 (STAT3) is the major signal transducer in the downstream of IL-6 and its involvement in VEGF gene regulation has already been postulated [18,42], we detected the expression level of VEGF in cell culture media from IL-6‐stimulated HPMCs. Our data showed that exogenous IL-6 promoted VEGF expression, and VEGF-abundant medium significantly increased endothelial cell tube formation, which was dependent on HDAC6 activity. This means IL-6 stimulates the mesothelial cell to secrete VEGF, which is the first step of peritoneal angiogenesis. And then VEGF induces proliferation, migration, vasoformation of vascular endothelial cells. Mechanistically, HDAC6 can regulate the recruitment and activation of STAT3 and subsequently promote its downstream signaling cascade [43,44]. Thus, HDAC6 inhibition may attenuate the peritoneal inflammatory-induced angiogenesis by blockade of the IL-6/STAT3/VEGF signaling axis. Furthermore, we found that HDAC6 expression was positively correlated with enhanced expression of IL-6 and VEGF in PD patients’ dialysis effluent. In this regard, HDAC6 might be a biomarker to identify PD patients who are under peritoneal inflammation and angiogenesis.

Except for the IL-6/STAT3/VEGF signaling pathway, Wnt/β-catenin is another important angiogenic pathway involved in peritoneal angiogenesis. Padwal et al. proved that Wnt/β-catenin signaling contributes to peritoneal membrane injury and regulates the level of VEGF in the presence of receptor tyrosine kinase-like orphan receptor (Ror2) [45]. Both of Dickkopf-related protein (DKK)-1 (inhibitor of Wnt) and ICG-001 (inhibitor of β-catenin) could attenuate peritoneal angiogenesis and reduce VEGF [11]. Interestingly, HDAC6 was reported to regulate β-catenin by deacetylation, leading to β-catenin phosphorylation and activation of downstream signal molecules [46]. Further research indicated that HDAC6-induced β-catenin deacetylation (K345) is essential for Wnt signal transduction [47]. So, we consider that HDAC6 may be associated with β-catenin deacetylation and activate Wnt/β-catenin-mediated angiogenic pathway during peritoneal angiogenesis.

Although this current research is designed and completed on the basis of our previous publication [33], it also presents plenty of novel findings and clinical values. Firstly, this study demonstrates that HDAC6 is highly expressed in both human peritoneum and PD effluent, and positively correlated with the expression of multiple cytokines/growth factors associated with peritoneal injury (i.e., TGF-β1, IL-6, VEGF, CA125), which suggests the potential role of HDAC6 as a noninvasive biomarker to estimate peritoneal status. Secondly, IL-6 exposure can stimulate PMCs to secrete VEGF, meaning that overexpression of IL-6 may be the causative factor on peritoneal angiogenesis especially for PD patients with peritonitis and micro inflammatory state. Moreover, HDAC6 is involved in this pathological process and contributes to the peritoneal neovascularization of endothelial cells. However, there are still some limitations and deficiencies in this study. For example, the number of patients in this group is not very more and we will amplify the cohort of this project in the future.

Collectively, this current study emphasizes and highlights the pathogenicity of HDAC6 in PD-related peritoneal injury in the clinic. We are the first to detect the level of HDAC6 in both peritoneum tissue and dialysate effluent from PD patients and suggest that HDAC6 has a good prospect for clinical application to be a noninvasive biomarker to estimate multiple peritoneal lesions. Moreover, inhibition of HDAC6 significantly decreases VEGF production through suppression of IL-6/STAT3 and the Wnt1/β-catenin signaling pathways. HDAC6 may become a therapeutic target on peritoneal angiogenesis for long-term PD patients.
